# Preparation and Hydrogen Absorption/Desorption of Nanoporous Palladium Thin Films

**DOI:** 10.3390/ma2042496

**Published:** 2009-12-23

**Authors:** Wen-Chung Li, Thomas John Balk

**Affiliations:** Department of Chemical and Materials Engineering, University of Kentucky, 177 F. Paul Anderson Tower, Lexington, KY 40506-0046, USA; E-Mail: wayne.li@uky.edu (W.C.L.)

**Keywords:** dealloying, nanoporous, palladium, hydridation

## Abstract

Nanoporous Pd (np-Pd) was prepared by co-sputtering Pd-Ni alloy films onto Si substrates, followed by chemical dealloying with sulfuric acid. X-ray diffractometry and chemical analysis were used to track the extent of dealloying. The np-Pd structure was changed from particle-like to sponge-like by diluting the sulfuric acid etchant. Using suitable precursor alloy composition and dealloying conditions, np-Pd films were prepared with uniform and open sponge-like structures, with interconnected ligaments and no cracks, yielding a large amount of surface area for reactions with hydrogen. Np-Pd films exhibited shorter response time for hydrogen absorption/desorption than dense Pd films, showing promise for hydrogen sensing.

## 1. Introduction

Interactions between hydrogen (H) and transition metals can lead to reversible formation of metal hydrides, with potential applications in H storage and purification. Pd interacts strongly with H and the two can exist as a solid solution hydride at room temperature, where H atoms occupy octahedral sites in the face-centered cubic (FCC) structure of Pd. For a H/Pd stoichiometric ratio between 0.01 and 0.6, the hydrides exist as immiscible α and β phases that both have FCC structures [1]. In addition, nanostructured Pd is believed to exhibit unique hydrogen absorption and desorption behavior, as well as a narrowing of the miscibility gap [2]. These effects are attributed to highly uncoordinated sites at the edges and corners of ledges on crystal surface planes [3,4]. In order to enhance its hydrogen absorption capability via size reduction of Pd clusters and increased surface area, Pd has been prepared primarily in two forms, namely nanoparticles [4,5,6] and thin films [7,8]. One difficulty with dispersed Pd nanoparticles is that they must be stabilized on a substrate for use in applications requiring large amounts of exposed surface area. To investigate the enhanced response of nanoscale Pd to hydrogen, Ding *et al.* [9] deposited Pd thin films on nanoporous anodic alumina templates for use as actuating coatings, and Mubeen *et al.* [10] tested Pd nanoparticles supported by carbon nanotubes for hydrogen sensing.

A blanket thin film of Pd deposited on a substrate offers improved mechanical stability, but limited surface area. Using standard thin film deposition techniques, it is difficult to simultaneously achieve both high surface area and good intrinsic mechanical integrity for the application of nanoscale Pd-based materials. In this study, nanoporous Pd (np-Pd) thin films were prepared under different dealloying conditions and were compared with respect to their structure and susceptibility to cracking.

Dealloying, or selective dissolution, is a reliable process for obtaining porous structures from an alloy containing two or more metallic elements. It is used to prepare nanoporous metals, with np-Au being a prime example. Dealloying can be performed with or without an applied potential. Electrochemical etching permits greater control over the dealloying rate and can achieve a lower amount of remnant alloying elements. On the other hand, free corrosion (no applied potential) is a simple approach that requires less processing equipment, can be implemented by more research groups, and also results in nanoscale porosity. Although dealloying to produce np-Au has been reported by several research groups [11,12,13], and although the mechanisms of dealloying have also been discussed and simulated in some detail [14,15], Pd has received very little attention, with two groups reporting the fabrication of bulk np-Pd [16,17,18], but no studies of thin film np-Pd. It is noted that, in addition to dealloying, other approaches can be used to achieve nanoscale porosity in a variety of materials, including Pd [19]. The current paper, however, focuses on dealloying to produce nanoporosity.

Similar to the use of AuAg alloys to produce np-Au, PdNi alloys were used as precursors to make np-Pd. Alloys of Pd and Ni are completely miscible at room temperature, which is helpful for obtaining a uniform np-Pd structure after dealloying. Another reason for choosing the Pd-Ni system is high selectivity between Pd and Ni during dissolution in sulfuric acid (Ni dissolves much more quickly). Additionally, there is a large difference in atomic size between Pd and Ni, and thus the lattice parameter varies significantly (~10%) between pure Pd and pure Ni. This size discrepancy allows the tracking of dealloying progress by x-ray diffraction (XRD), which is not possible with the Au-Ag system due to the very similar lattice parameters of Au and Ag. The approach has been used for tracking phase changes in Ni-Cu during dealloying [20]. The current study focused on the preparation of np-Pd thin films by dealloying, including identification of the point at which dealloying was completed, and on the microstructure and hydrogen absorption/desorption behavior of np-Pd films.

## 2. Results and Discussion

Sulfuric acid was chosen as the dealloying solution due to its high selectivity between Pd and Ni. To assess the selectivity, films of pure Pd (100 nm thick) and Ni (60 nm thick) were deposited on Si substrates, using the same processing parameters used for deposition of PdNi alloy precursor films. Film thickness was measured using contact profilometry. The pure Pd and Ni films were immersed in both concentrated (98% stock solution) and dilute (25%) sulfuric acid for 70 h. Immediately upon immersion in concentrated and dilute H_2_SO_4_, the Ni film surface began to generate bubbles. These bubbles were attributed to the evolution of hydrogen gas, which would result from the oxidation of Ni and reduction of H^+^ ions in the acid solution. In contrast, no bubbles were observed on the surface of the pure Pd film. After immersion in the acid solutions for 70 h, the Ni film was completely dissolved, except for isolated patches of black residue that did not adhere well to the substrate. The Pd film, however, was intact after 70 h and had not undergone any color change. Additionally, the thickness of the Pd film, measured after removal from the acid solutions, was unchanged at 100 nm. These observations, together with the fact that Pd is more noble than Ni, indicate that Ni should be preferentially removed from the PdNi precursor, *i.e.,* Pd dissolution is expected to be minimal. While dissolved Ni should exist in solution as Ni^2+^ (NiSO_4_ is highly soluble in water), PdSO_4_ is only slightly soluble in water and thus may be incorporated into Pd ligaments within the np-Pd film. To check for such impurities, np-Pd film composition was measured using energy dispersive X-ray spectroscopy (EDS). Pd, Ni and Si were detected in all films, as were lower amounts of carbon and oxygen, presumably from residual organic solvent used for rinsing. However, no sulfur signal was detected in any film, suggesting that np-Pd contained either minimal or no PdSO_4_. Thus, even if Pd was dissolved during dealloying, it does not appear that impurity byproducts were incorporated in the np-Pd films.

### 2.1. Tracking the Extent of Dealloying

**Figure 1:**
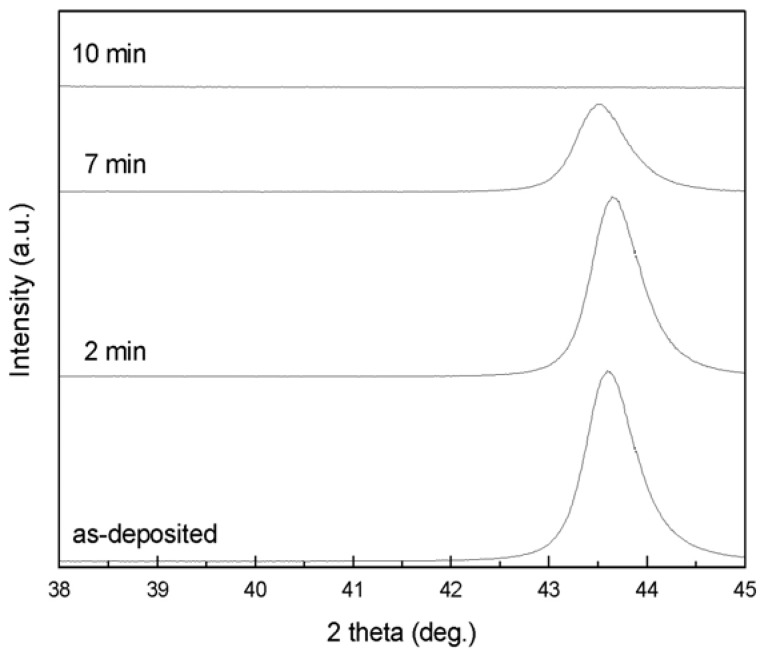
Evolution of dealloying in a 300 nm PdNi film immersed in concentrated (98%) sulfuric acid, as tracked by the integrated intensity of the PdNi alloy peak at ~43.6° in XRD scans. The alloy peak is still relatively strong after dealloying for 7 min, but disappears completely by 10 min dealloying time.

Dealloying of a 300 nm PdNi alloy film in concentrated sulfuric acid (98% stock solution) for 0 to 10 min was tracked by XRD, as illustrated in Figure 1. Based on the lattice parameters of pure Pd and Ni, their (111) peaks should appear at 40.1° and 44.5°, respectively. XRD scans for each specimen were calibrated against the Si (400) peak position, which should lie at 69.1°. In Figure 1, a diffraction peak at ~43.6° in each scan (except for the scan of the PdNi film dealloyed for 10 min) is attributed to the Ni-rich PdNi precursor alloy. Integrated intensity of the alloy peak decreased as dealloying progressed, until the peak eventually disappeared. Final disappearance of the peak, which occurred abruptly and rapidly, indicated that the PdNi alloy phase could not be detected by XRD, even though remnant Ni was detected by EDS. According to the peak shift relative to the expected peak positions for pure Ni and pure Pd, the Pd concentration of the as-deposited alloy film was ~19 at %, in agreement with the value of 18 at % measured by EDS. During dealloying, Pd concentration in the PdNi alloy varied slightly from 19 at % to 22 at %, as determined from XRD scans. For some dealloyed films, a new peak at 40.1°, corresponding to np-Pd, was not observed in XRD scans, e.g., Figure 1. This may be due to the crystallite size effect, as calculations using the Scherrer equation suggest that np-Pd ligament sizes could cause significant peak broadening. A ligament width of 10 nm would broaden the np-Pd peak by 2.3° full-width half-maximum (FWHM), while a 5 nm ligament width would cause broadening of 4.6° FWHM. Additionally, dealloying removes ~75% of the atoms from the precursor alloy, which would lower the peak intensity by a factor of ~2.4 (after accounting for the different atomic scattering factors of Ni and Pd). The combined effect could thus cause a reduction in XRD peak intensity of 90% to 95%. XRD scans appear to be able to detect the disappearance of the precursor phase, but not the appearance of np-Pd if ligament width is sufficiently small. 

**Figure 2:**
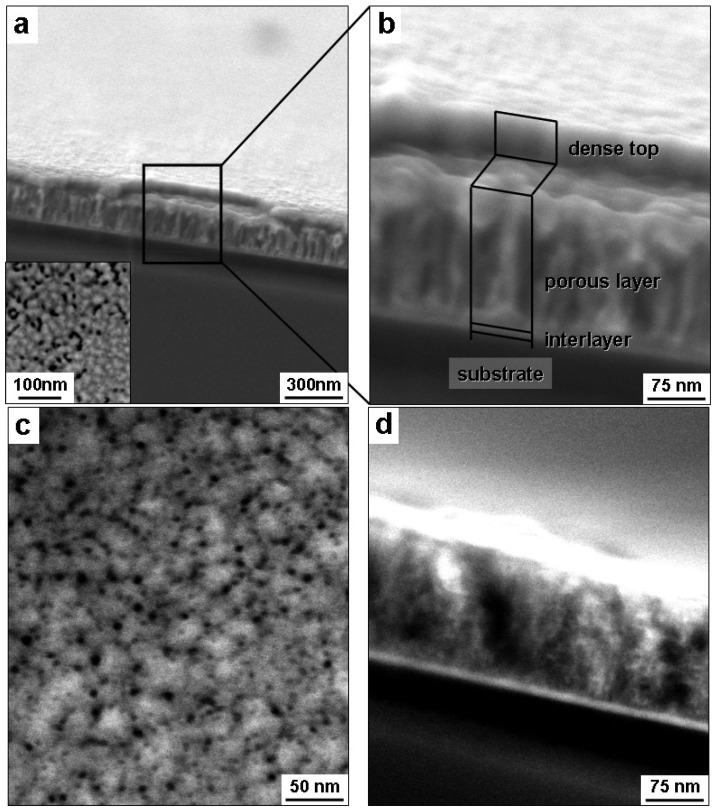
(a) and (b) Cross-sectional SEM micrographs of a 300 nm PdNi film dealloyed by 98% sulfuric acid for 2 min (partial dealloying), which produced a multilayered structure: Si substrate, amorphous silicon nitride coating, Ta and Pd interlayers, np-Pd film, and a dense top layer. A plan view observation is shown in the inset of (a). (c) Plan view and (d) cross-sectional SEM micrographs of the film dealloyed for 10 min, which resulted in a nanoporous structure with no dense top layer.

Cross-sectional and plan view scanning electron microscope (SEM) images of the 300 nm PdNi films dealloyed with 98% sulfuric acid for 2 and 10 min are shown in Figure 2. In Figure 2a, cross-sectional micrographs (with the sample tilted by 10°) of the film dealloyed for 2 min exhibited a multilayered structure: from bottom to top, one can see the Si substrate, ~50 nm amorphous silicon nitride, ~15 nm total thickness of Ta and Pd interlayers, 185 nm porous PdNi layer, and a 45 nm top layer. The top layer above the porous PdNi film exhibited a uniform thickness ~45 nm. As seen at higher magnification of an area where the top layer was partially peeled away (Figure 2b), this top layer sat on a rougher porous PdNi layer. The porous PdNi layer exhibited elongated pores, with dimensions ~20 nm wide and 150 nm long, which were oriented roughly perpendicular to the Si substrate. The inset in Figure 2a is a plan view observation of the partially dealloyed PdNi film. In this inset, white clusters ~20 nm in diameter were distributed over most of the surface, except for a number of dark areas, and the cluster/hole size was the same as the grain size in as-deposited precursor films.

Figure 2c and d show plan view and cross-sectional SEM images of the microstructure of the PdNi film dealloyed for 10 min (at which point the alloy phase was no longer detected by XRD). Pd clusters 15–30 nm wide were surrounded by pores ~5 nm in diameter. The cross-sectional image shown in Figure 2d reveals a porous structure through the film thickness, but the top layer shown in Figure 2a and b has disappeared; moreover, pores are less than 10 nm in diameter, in agreement with plan view observations (Figure 2c). The thickness of the dealloyed film decreased by ~45%, from 300 nm to 165 nm. Sun and Balk [21] reported that the thickness of 25Au-75Ag alloy films decreased by ~20% during dealloying to form np-Au, much less than the contraction observed here for np-Pd. The final thickness of np-Pd (165 nm) is believed to result from contraction of the porous PdNi layer shown in Figure 2b (185 nm, not including the 45 nm dense top layer). The top layer shown in Figure 2b peeled away from the underlying film at longer dealloying times, and this appears to be responsible for the rapid disappearance of the PdNi alloy peak from XRD scans.

**Figure 3:**
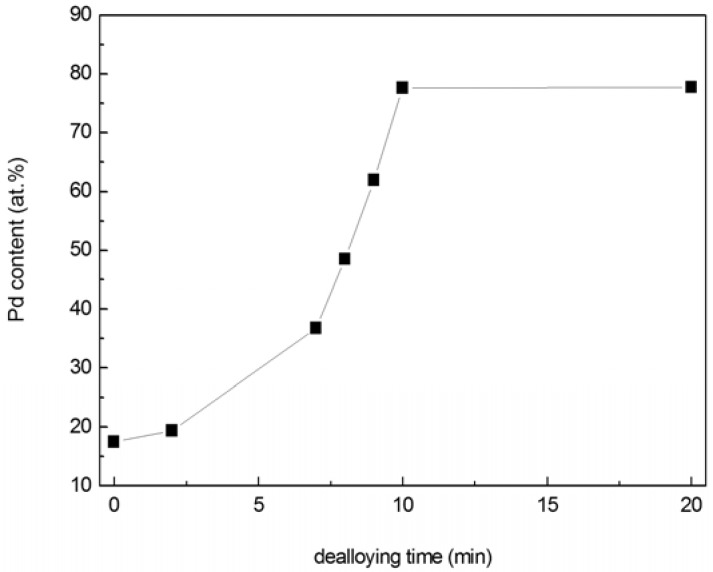
Changes in Pd content of PdNi alloy films at dealloying times from 0 to 20 min. Pd content increases rapidly between 7 and 10 min dealloying time, and exhibits a plateau at ~78 at % beyond this point.

Composition of the thin film varied during dealloying and was determined using EDS, as shown in Figure 3. The EDS data displayed in Figure 3 represent compositions calculated from Pd and Ni only. Typically, the EDS signal also contained contributions from Si and N (the Si substrate was coated with an amorphous silicon nitride layer), as well as C and O (hydrocarbon residue from ethanol rinsing). The combined amount of C and O, for analyses that included Pd/Ni/C/O (but not Si/N, as these were confined to the substrate), was ~5 at % or less. Since C and O were assumed to reside on the np-Pd ligament surfaces, they were not included in the compositions presented in Figure 3. Total Pd content increased slightly during the first 2 min of dealloying, from ~15 to ~19 at %. As dealloying time progressed from 7 min to 10 min, however, Pd concentration increased rapidly from 37 to 78 at %, after which it exhibited a plateau at ~78 at %. The slight change in Pd content during the first 2 min of dealloying is consistent with the slight decrease in integrated intensity of the alloy diffraction peak shown in Figure 1, *i.e.,* a small portion of precursor PdNi was dealloyed to form np-Pd and thus the total Ni content decreased. This is also consistent with the formation of porosity within the first 2 min of dealloying (see Figure 2a-b). During continued dealloying (to 7 min), total Pd content increased significantly (Figure 3) and the alloy diffraction peak experienced a concomitant decrease in integrated intensity (Figure 1). Starting at 10 min dealloying time, the np-Pd film exhibited a plateau in Pd content, suggesting that the dealloying process had run its course. Given that a post-dealloying Ni content of 22 at % was detected by EDS, the remnant Ni may reside in the np-Pd ligament structure. As seen in Figure 1, the alloy diffraction peak disappeared completely by 10 min dealloying time, and SEM (Figure 2d) revealed a fully porous film with no dense top layer. The lack of a np-Pd diffraction peak in Figure 1 is typical of this material, for which diffraction peaks are low or non-existent. This may be due to the small diameter of ligaments, which cause extreme peak broadening and may be susceptible to rotation out of the diffracting condition.

### 2.2. Effect of Dealloying Rate on Nanoporous Structure

**Figure 4:**
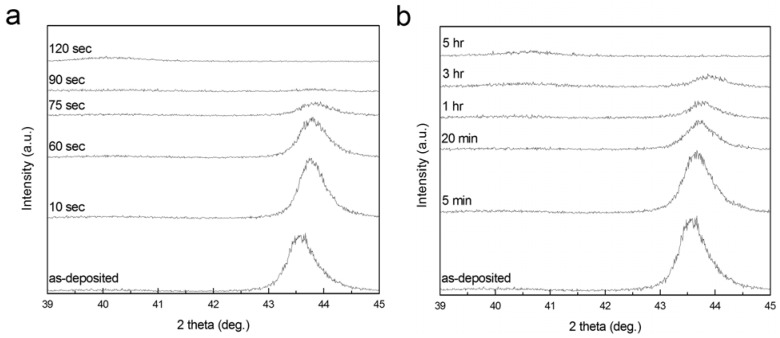
Evolution of 90 nm PdNi films dealloyed in (a) 98% and (b) 25% sulfuric acid, as tracked by the integrated intensity of the PdNi alloy peak at ~43.6° in XRD patterns. During dealloying, the PdNi peak shifted to progressively higher angles, perhaps due to changes in film stress. In XRD scans of both films at long dealloying times, a low, broad peak observed near the expected Pd position of 40.1° is likely due to np-Pd.

To investigate the effect of dealloying rate on microstructure of np-Pd, 90 nm PdNi alloy films were dealloyed in concentrated (98% stock solution) and dilute (25%) sulfuric acid, and the PdNi alloy peak was tracked by XRD, as shown in Figure 4. Figure 4a presents a series of XRD scans of a PdNi alloy film in the as-deposited state, and after dealloying (of the same sample) for 10 sec to 120 sec in concentrated sulfuric acid. Similar to dealloying of the 300 nm PdNi film shown in Figure 1, the peak attributed to the precursor alloy at ~43.6° was observed in each scan, except for a dealloying time of 120 sec. Pd concentration in the PdNi alloy, as estimated from the PdNi alloy peak position, was ~19 at % before dealloying. This peak shifted to higher angles during dealloying, indicating that the interplanar spacing of the {111} diffracting planes decreased. This is consistent with changes in the biaxial film stress measured at various stages of dealloying: stress was initially highly compressive (−1.4 GPa), but relaxed to −28 MPa (compressive) at 120 sec. This net tensile change in film stress would cause the spacing between diffracting planes to decrease. Figure 4b shows a series of XRD scans for a second PdNi film, as-deposited and subsequently dealloyed for 5 min to 5 h in dilute sulfuric acid. The PdNi alloy peak disappeared after 5 h dealloying time, indicating that a disproportionately longer dealloying time was required when the sulfuric acid concentration was decreased from 98% to 25%. Dealloying in the dilute acid was at least two orders of magnitude slower than in the concentrated acid.

**Figure 5:**
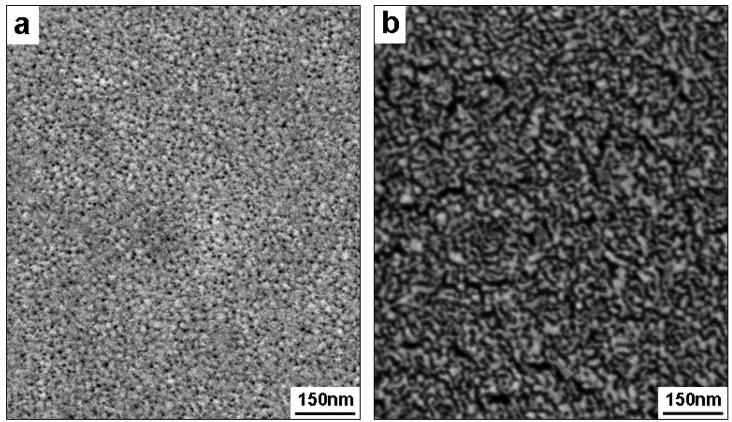
Microstructure of np-Pd obtained from 90 nm PdNi films dealloyed in (a) concentrated (98%) and (b) dilute (25%) sulfuric acid solutions. The films exhibit particle-like and sponge-like structures, respectively, with cluster/ligament size on the order of 10 nm.

As shown by the plan view SEM micrographs in Figure 5, np-Pd films processed in concentrated *versus* dilute sulfuric acid exhibited significant microstructural differences. When dealloyed in concentrated sulfuric acid, np-Pd consisted of 10–15 nm wide, particle-like Pd clusters surrounded by pores 5–10 nm in diameter (see Figure 5a). However, when processed in dilute sulfuric acid, np-Pd exhibited a sponge-like interconnected structure with elongated Pd ligaments, 10−15 nm long and ~10 nm wide, as shown in Figure 5b. However, randomly oriented cracks as large as ~175 nm long and ~20 nm wide were observed throughout the np-Pd film after etching in dilute sulfuric acid. During dealloying, the removal of a majority of atoms tends to cause significant volume contraction [22] and can also shift the film stress toward more tensile values. Cracking tends to relax any film stress that is present, as does the rearrangement of Pd atoms during ligament coarsening. All of these effects would cause the initially compressive stress in PdNi films to relax toward zero, as was observed here.

### 2.3. Effect of Pd Concentration on Nanoporous Structure

**Figure 6:**
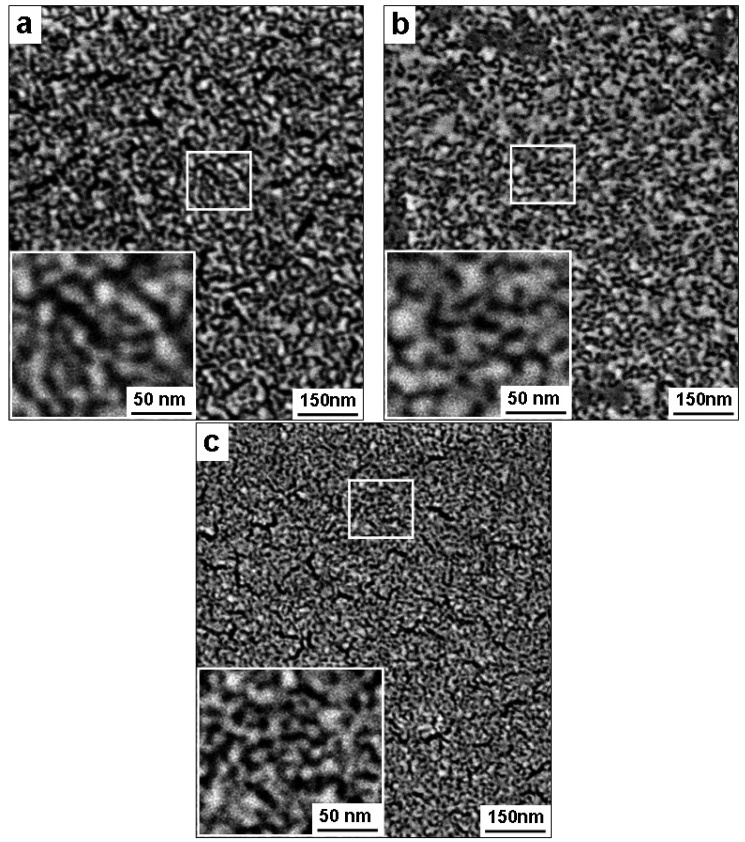
Microstructure of np-Pd produced from 90 nm PdNi precursor alloy films with (a) 18 at % Pd, (b) 22 at % Pd, and (c) 25 at % Pd. All films were dealloyed in 25% sulfuric acid and exhibited an open, sponge-like structure with ligaments and pores smaller than 10 nm. Cracks were observed in the films obtained from 18 at % Pd and 25 at % Pd precursor films, but not in the 22 at % Pd film shown in (b).

Composition of the PdNi precursor alloy was varied to investigate the effect of Pd content on the final structure of np-Pd films. The desired microstructure for these films was a 3-D interconnected network of Pd ligaments and open pores, with no film cracks. The initial Pd content was 18, 22 or 25 at % for PdNi alloy films with a thickness of 90 nm. All films were dealloyed for 5 h in dilute (25%) sulfuric acid. In Figure 6, the three types of thin film np-Pd exhibited similar porous structures, but with different degrees of cracking. The Pd ligaments seen in Figure 6a and Figure 6b (18 at % and 22 at % Pd precursor films, respectively) were ~10 nm wide and 10–20 nm long. In contrast, the ligaments imaged in Figure 6c (from the 25 at % Pd precursor alloy) were much finer, *i.e.,* ~5 nm wide and 10–15 nm long. Additionally, Figure 6b shows that the np-Pd film was crack-free, whereas cracks were apparent in Figure 6a and Figure 6c. Maximum crack dimensions were ~175 nm long and ~20 nm wide in Figure 6a, *versus* ~125 nm long and ~15 nm wide in Figure 6c. It appears that film cracking could be mitigated by setting the initial Pd content at 22 at %. This agrees with earlier studies that found optimum precursor compositions for producing crack-free films. For example, Lu *et al.* [13] proposed that cracking in np-Au films can be avoided by increasing the Au content in the precursor alloy to an intermediate level. Additionally, Sun *et al.* observed significant cracking in np-Au films obtained from 25 at % Au films [21], but they found no cracking when the precursor Au content was increased to 30 at % [23]. It should be noted, however, that np-Pd microstructures characterized in the current study may have been affected by stress fields generated by the cleaved edges of Si wafer pieces. Although crack-free films of nanoporous metals are achieved with an optimum precursor alloy composition, film cracks typically appear when there is too little noble metal in the precursor (too much noble metal leads to retained islands of the alloy). The observation of cracks in np-Pd films produced from both 18 and 25 at % Pd precursors could be due to varying stress fields. Nonetheless, the absence of cracks in Figure 6b suggests that 22 at % Pd is a suitable precursor alloy composition for producing np-Pd films. 

### 2.4. Hydrogen Absorption/Desorption Behavior of np-Pd Films

**Figure 7:**
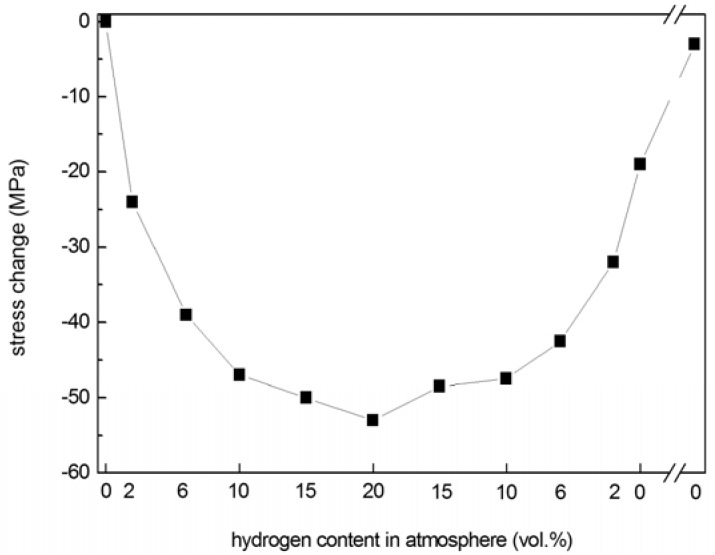
Hydrogen absorption/desorption behavior of a crack-free, 90 nm np-Pd film, produced from a precursor alloy with 22 at % Pd. Ambient hydrogen content was increased from 0% to 20% (by volume) and then decreased back to 0%. The np-Pd film stress became increasingly compressive with higher hydrogen content, and remained at a significant level after removal of hydrogen from the atmosphere. However, after 10 min in flowing nitrogen, stress returned to approximately the same value as that measured for the as-dealloyed film before exposure to hydrogen.

In Pd hydrides, H atoms occupy octahedral interstitial sites in the Pd matrix. During hydridation and dehydridation, which cause reversible transformation between pure Pd and hydride phases, the resulting lattice expansion and contraction lead to stress changes in the film that can be used to track hydrogen absorption and desorption. The volume occupied by equivalent unit cells in each lattice is ~11% higher in β-PdH_x_ (x = ~0.6 at 1 atm) than in pure Pd [24,25]. In the current study, the stress of np-Pd films exposed to varying amounts of atmospheric hydrogen was measured in order to evaluate hydrogen sensitivity. Np-Pd was produced from 90 nm PdNi precursor films (22 at % Pd) dealloyed in dilute sulfuric acid for 5 h. Hydrogen content was increased from 0 to 20 vol % in 5 steps and then sequentially decreased to 0 vol % using the opposite sequence. After each step, hydrogen content was held constant for 3 min and stress measurements were taken every 20 sec. The film stress at each step was taken as the average of all measurements at a given hydrogen content, and the stress change (*versus* the initial stress of −28 MPa, measured without hydrogen present) was calculated. As seen in the plot of hydrogen absorption/desorption behavior in Figure 7, film stress became more compressive with increasing hydrogen content, leading to a maximum change of −54 MPa in 20 vol % hydrogen.

As the amount of atmospheric hydrogen was decreased, film stress relaxed toward its original value. However, the stress did not immediately recover when hydrogen was reduced to 0 vol %, but maintained a residual stress change of −20 MPa (compression). After 10 min, the residual compressive stress had relaxed nearly to the original stress (−4 MPa residual change). This lag in stress recovery may be due to a portion of H atoms being trapped in (or slowly released from) the np-Pd ligaments.

**Figure 8:**
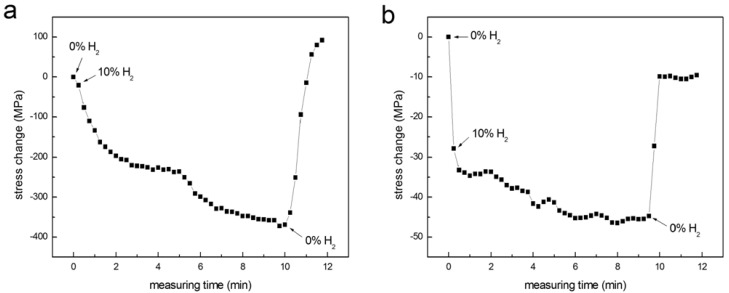
Stress changes measured in (a) 100 nm dense Pd film and (b) 90 nm np-Pd film, in an atmosphere where hydrogen content was rapidly changed from 0% to 10% (by volume). The np-Pd film stress reached a plateau six times faster than the dense Pd film stress (which also exhibited an intermediate plateau). The points at which hydrogen flow was switched on/off are indicated on each plot.

The response times of thin films of Pd and np-Pd to hydrogen were also tested by abruptly switching between hydrogen contents of 0 and 10 vol % in a flowing, mixed nitrogen-hydrogen atmosphere. Stress in the 90 nm np-Pd film was measured at 15 sec intervals during the test. Results were compared with a fully dense, 100 nm thick Pd film, shown in Figure 8. The initial stress in the dense Pd and np-Pd films were +85 and +6 MPa, respectively. When the hydrogen content was switched to 10 vol %, stress immediately began to shift to a compressive state. The dense Pd film exhibited a gradual change in stress to −287 MPa, with two plateau regions that were separated at a time of ~5 min. The Pd film reached the first stress plateau (−140 MPa) at ~3 min. In contrast, the np-Pd film stress changed rapidly when the hydrogen content was switched from 0 to 10 vol %, initially settling at −28 MPa within 30 sec and later shifting to a plateau at −41 MPa after ~8 min. Although the magnitude of stress change associated with the first plateau was six times higher in Pd *versus* np-Pd, the time required to reach this plateau was also six times longer in Pd *versus* np-Pd. Plotting both curves on the same axes, and using relative stress change as the vertical axis, confirmed that the np-Pd film exhibited a shorter time to stress saturation. When the hydrogen content was switched back to 0 vol %, the np-Pd film displayed a much shorter response time than the dense Pd film, and stress relaxed nearly to the original value within 30 sec. Overall, np-Pd exhibited more rapid stress changes upon exposure to hydrogen than did the dense Pd film. This is likely due to the higher amount of surface area and shorter diffusion length in individual Pd ligaments. In the np-Pd film, H atoms are more easily absorbed at the ligament surfaces and can penetrate into the interior regions of ligaments.

## 3. Experimental Section

PdNi alloy films were magnetron co-sputtered onto Si substrates (CrysTec GmbH, Berlin, Germany) using an Ar plasma in a high vacuum system (base vacuum better than 1 × 10^−6^ Pa) at room temperature. Substrates were (100)-oriented, 180 μm or 380 μm thick, single crystalline Si wafers coated with 10 nm amorphous silicon oxide and 50 nm amorphous silicon nitride. The amorphous silicon nitride surface was cleaned, prior to film deposition, by substrate biasing at 35 W (RF power) for 1.5 min. 10 nm Ta and 10 nm Pd films were sputtered as interlayers to improve adhesion between the final np-Pd film and the substrate. Deposition of PdNi alloy films with different compositions was achieved by varying the sputtering power for the Pd and Ni targets. Three compositions of the PdNi precursor alloy were produced: 18, 22 and 25 at % Pd. Composition was controlled by changing the sputtering power applied to Pd and Ni targets. Substrate biasing (35 W, RF power) was applied during alloy film deposition, resulting in 90 nm and 300 nm films with a purely {111} film texture (as determined by XRD). Film thickness was subsequently measured by surface profilometry (Veeco Dektak 6M Stylus Profiler) and was found to differ from the expected thickness (based on known deposition rate) by less than 4%. 

The as-deposited alloy films were dealloyed in either concentrated (98 vol % stock solution) or dilute (25 vol %) sulfuric acid, which can remove Ni atoms from the alloy phase and transform Pd into a nanoporous structure. Acid solutions were all at room temperature and dealloying was performed for times ranging from 2 min to 5 h. The longer times were required for the dilute etchant. For dealloying in concentrated sulfuric acid, samples were held with tweezers and continuously moved within the solution. For dilute-acid dealloying, samples were simply immersed in the etching solution; at ~30 min intervals, samples were gently agitated by holding them with tweezers and moving them in the solution. During dealloying, the film was observed to change color from metallic silver to dark brown, which coincided with the disappearance of the {111} PdNi precursor alloy peak and with the onset of the plateau in maximum Pd content of dealloyed np-Pd.

As-dealloyed specimens were rinsed and immersed in ethanol (95%) for at least 1 h to remove remnant sulfuric acid in the nanoporous structure. For tracking the progress of dealloying, XRD (Siemens model D500) was used to determine the presence of the PdNi alloy phase in the films. Consistent parameters were used for all specimens: 38°−45° scan range, 0.01° step size, and either 0.1°/min or 4°/min scan speed. The position of the Si (400) peak was measured for every sample, to determine and correct any peak shift in the XRD scans. Microstructure of np-Pd films was observed in plan view with a scanning electron microscope (Hitachi S900) operated at 3 kV in secondary electron mode. Composition of the as-deposited and dealloyed PdNi films was determined by energy dispersive X-ray spectroscopy (EDS, Hitachi S3200 SEM). For XRD, SEM and EDS characterization, samples ~5 mm × ~5 mm in size were cut from pieces of film-coated wafer prior to dealloying. Stress changes during hydrogen absorption/desorption of np-Pd thin films were measured with a wafer curvature system (FLX-2320-S, Toho Technology Co.) equipped with an atmosphere-controlled chamber. Specimens used for stress measurement were prepared by dealloying a PdNi precursor alloy film on a 3-inch Si wafer. Ambient hydrogen content was regulated by mixing zero-grade hydrogen (99.9% purity) and ultrahigh purity nitrogen (99.999%), with each gas controlled by a separate flow controller. Hydrogen content was adjusted from 0 to 20 vol % in 5 steps and then sequentially decreased to 0 vol % using the opposite sequence. During each step, hydrogen content was held for 3 min and stress measurements were taken every 20 sec. The film stress at each step was taken as the average of all measurements at a given hydrogen content, and the stress change was calculated relative to the film stress measured with no hydrogen in the atmosphere. Stress was measured at 20 sec intervals during hydrogen absorption/desorption. During measurements of response time, when hydrogen flow was simply turned on/off and not changed in small steps, stress was measured at 15 sec intervals. All stress measurements were made at room temperature.

## 4. Conclusions

Np-Pd films were prepared by dealloying PdNi alloy films in sulfuric acid. Evolution of dealloying in 90 nm and 300 nm PdNi alloy films was monitored with XRD, by tracking the position and integrated intensity of the PdNi alloy peak. PdNi alloy films dealloyed in dilute sulfuric acid yielded np-Pd films with a sponge-like and interconnected ligament structure, with ligaments/pores ~10 nm in size. Film cracking was observed in np-Pd films obtained from precursor alloys with high and low Pd contents, but an intermediate composition (22 at % Pd) resulted in crack-free films. Hydrogen absorption/desorption behavior of np-Pd was evaluated by measuring changes in film stress. Crack-free films are important for such measurements, since cracks would lower the magnitude of film stress changes caused by H absorption. The fine pore/ligament structure and high amount of surface area resulted in high sensitivity and rapid response times upon hydrogen exposure, suggesting that np-Pd may be a promising material for hydrogen sensing.
